# Decreased secretion of adiponectin through its intracellular accumulation in adipose tissue during tobacco smoke exposure

**DOI:** 10.1186/s12986-015-0011-8

**Published:** 2015-05-09

**Authors:** Mingzhen Li, Chunjun Li, Yu Liu, Yan Chen, Xiangdong Wu, Demin Yu, Victoria P Werth, Kevin Jon Williams, Ming-Lin Liu

**Affiliations:** Section of Endocrinology, Diabetes & Metabolic Diseases, Temple University School of Medicine, Philadelphia, PA USA; Department of Dermatology, Perelman School of Medicine, University of Pennsylvania, Philadelphia, PA USA; Philadelphia Veterans Administration Medical Center, Philadelphia, PA USA; The Metabolic Disease Hospital, Tianjin Medical University, Tianjin, China; Department of Molecular and Clinical Medicine, Sahlgrenska Academy at the University of Gothenburg, Gothenburg, Sweden

**Keywords:** Adiponectin, Tobacco smoke, Adipocytes

## Abstract

**Background:**

Cigarette smoking is associated with an increased risk of type 2 diabetes mellitus (T2DM). Smokers exhibit low circulating levels of total adiponectin (ADPN) and high-molecular-weight (HMW) ADPN multimers. Blood concentrations of HMW ADPN multimers closely correlate with insulin sensitivity for handling glucose. How tobacco smoke exposure lowers blood levels of ADPN, however, has not been investigated. In the current study, we examined the effects of tobacco smoke exposure in vitro and in vivo on the intracellular and extracellular distribution of ADPN and its HMW multimers, as well as potential mechanisms.

**Findings:**

We found that exposure of cultured adipocytes to tobacco smoke extract (TSE) suppressed total ADPN secretion, and TSE administration to mice lowered their plasma ADPN concentrations. Surprisingly, TSE caused intracellular accumulation of HMW ADPN in cultured adipocytes and in the adipose tissue of wild-type mice, while preferentially decreasing HMW ADPN in culture medium and in plasma. Importantly, we found that TSE up-regulated the ADPN retention chaperone ERp44, which colocalized with ADPN in the endoplasmic reticulum. In addition, TSE down-regulated DsbA-L, a factor for ADPN secretion.

**Conclusions:**

Tobacco smoke exposure traps HMW ADPN intracellularly, thereby blocking its secretion. Our results provide a novel mechanism for hypoadiponectinemia, and may help to explain the increased risk of T2DM in smokers.

## Findings

### Introduction

Over 1.3 billion people smoke worldwide, and even more are exposed to second-hand smoke. Smokers often exhibit impairments in insulin-mediated glucose handling and an increased incidence of type 2 diabetes mellitus (T2DM) [1]. Smoking cessation improves these conditions [2]. Nevertheless, mechanisms by which smoking impairs insulin-stimulated glucose metabolism and increases T2DM are still unclear.

Adiponectin (ADPN), an insulin-sensitizing adipokine secreted from adipose tissue, circulates in three different multimeric forms – namely, trimers (low molecular-weight, LMW), heximers (medium molecular-weight, MMW), and larger multimers (high molecular-weight, HMW) [3]. HMW ADPN is considered the most active form of ADPN [4,5], and peripheral insulin sensitivity for handling glucose closely correlates with blood levels of HMW ADPN [3-6]. Smokers exhibit low blood levels of total [6,7] and HMW [8] ADPN, while smoking cessation has been shown to restore blood levels of total ADPN [9,10]. Similarly, in mice, tobacco smoke exposure decreases blood ADPN [11]. How tobacco smoke exposure lowers blood levels of ADPN, however, has not been investigated [10]. In the current study, we sought to determine the potential effects of tobacco smoke exposure on ADPN multimerization and secretion, as well as the relevant intracellular molecular mediators in these processes.

## Methods

### Materials

We purchased polyclonal antibodies against mouse adiponectin and ERp44 (endoplasmic reticulum [ER] resident protein of 44 kDa) from Cell Signaling. Monoclonal antibodies against Ero1 L-α (ER oxidoreductase 1-Lα) and GAPDH, as well as secondary antibodies (anti-rabbit and anti-mouse IgG horseradish peroxidase conjugates), were from Santa Cruz. The antibody against DsbA-L (disulfide-bond A oxidoreductase-like protein) was from Abcam. Tobacco smoke extract (TSE, 100%) with water-soluble components was prepared by using a Kontes gas-washing bottle to bubble mainstream smoke from research cigarettes through serum-free, phenol red-free RPMI media containing 0.2% BSA (RPMI/BSA), followed by filtration (0.22 μm) and standardization according to their absorbance at 320 nm, as we previously published [12,13].

### Cell culture, preparation of primary mouse adipocytes, and TSE exposure

Murine 3T3-L1 preadipocytes (ATCC) were cultured and differentiated into adipocytes as described [14]. Briefly, two days after reaching 100% confluence, the cells were stimulated for an additional two days with FBS/DMEM containing 100 nM insulin, 0.5 M IBMX, 0.25 μM dexamethasone, and 1 μM rosiglitazone. Cells were then maintained in FBS/DMEM medium with 100 nM insulin for another 2 days to differentiate into mature adipocytes with fat droplets. Cells were serum-starved for 3 h in DMEM containing 0.2% BSA, followed by exposure to 0–1.5% TSE for 0-20 h. Primary mouse adipocytes were prepared as described [15]. Briefly, epididymal adipose tissue from wild-type C57BL/6 mice (Jackson Laboratory) was placed in pre-warmed DMEM with 10% FBS and penicillin/streptomycin, and then minced into 5–10 mg pieces. Minced tissue fragments were filtered through a nylon mesh (350-μm pore size) and washed with DMEM. Then 200-300 mg of minced, filtered tissue was placed into 1 ml DMEM with 0.2% BSA and penicillin/streptomycin for 18 h before being treated without or with 1.5% TSE for an additional 20 h. At the end, the supernatants were collected and the explants were lysed for ADPN immunoblots.

### TSE exposure of wild type mice

To mimic the effects of tobacco smoke exposure on a non-respiratory organ with well-controlled dosing, we followed the recently established methodology of intraperitoneal administration of TSE [16-18]. Wild-type mice were injected intraperitoneally in the lower left quadrant of the abdomen with 400 μl of pre-warmed, filtered TSE diluted to 20% strength in RPMI-1640 (this amount of TSE is equivalent to smoking 2 packs of cigarettes for a 60-kg person) or RPMI-1640 alone (control) on days 1, 3, 5, 8, and 10 [16]. Twenty-four hours after the final injection, the mice were euthanized by an overdose of pentobarbital. We collected whole blood by cardiac puncture and then epididymal adipose tissue from the lower right abdominal quadrant, away from the application sites. All animal protocols were approved by the Institutional Animal Care and Use Committee of the Philadelphia Veterans Administration Medical Center.

### Immunoblots

Immunoblots were performed as described in our previous publications [12,13]. For detection of adiponectin oligomers and multimers, cells were lysed in non-reducing lysis buffer and loaded onto a gel without boiling.

### Determination of adiponectin concentration

Total adiponectin concentrations in conditioned media from control and TSE-treated 3T3-L1 adipocytes and in plasma from control and TSE-treated mice were measured by ELISA for mouse ADPN according to the manufacturer’s instruction (BioVendor). 

#### Data analysis

All column and line graphs depict mean ± SEM of data that passed tests for normality. Comparisons amongst three or more groups were performed using one-way analysis of variance (ANOVA), followed by pairwise comparisons using Student-Newman-Keuls (SNK) test, with p < 0.05 considered significant. Comparisons between two groups used Student’s unpaired, two-tailed t-test.

## Results and discussion

### Tobacco smoke exposure decreases secretion of ADPN while inducing its intracellular accumulation

**Figure 1:**
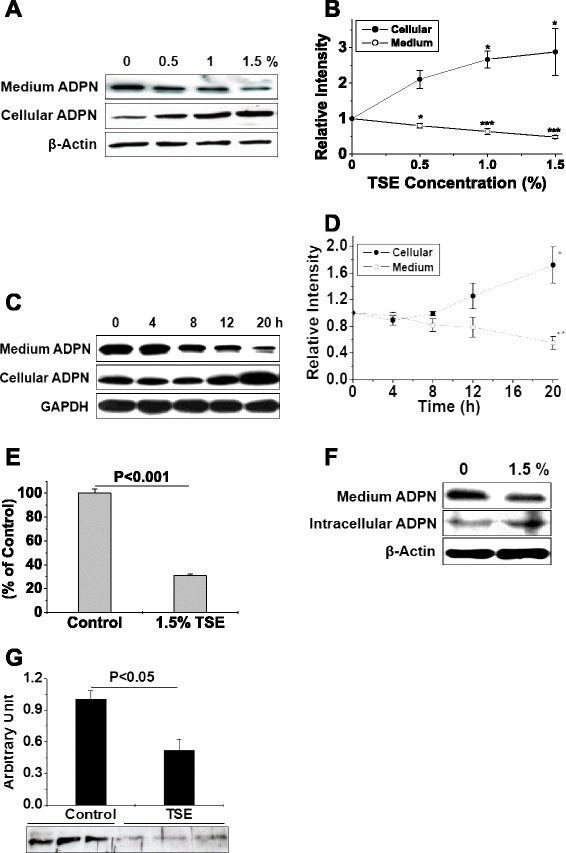
Tobacco smoke exposure decreases secretion of ADPN while inducing its intracellular accumulation. Panels **A,B**: Representative immunoblots (**A**) and summary statistics (**B**) for dose-dependent effects of TSE on ADPN accumulation in conditioned medium and in cellular homogenates of 3T3-L1 adipocytes during a 20-h incubation. Panels **C,D**: Representative immunoblots (**C**) and summary statistics (**D**) of the time course of the effects of TSE on ADPN accumulation in conditioned medium and in cellular homogenates of 3T3-L1 adipocytes exposed to 1.5% TSE for 0-20 h. Panel **E**: ADPN concentrations measured by ELISA in the culture supernatants of 3T3-L1 adipocytes exposed for 20 h to 0 (control) or 1.5% TSE . Panel **F**: Immunoblots of ADPN in conditioned medium and in cellular homogenates of primary mouse adipocytes treated without or with 1.5% TSE for 20 h. Panel **G**: Immunoblots of total plasma ADPN in mice after RMPI (Control) or TSE injections. Panels **B, D, E, G**, n = 3-5. In panels **B** and **D**, *P* < 0.01 by ANOVA of all cellular values, and *P* < 0.01 by ANOVA of all medium values. *P < 0.05, **P < 0.01, ***P < 0.001 vs. control values (0% TSE or t = 0) by the SNK test. In panels **E** and **G**, Student’s t-test was used.

We found that TSE exposure caused dose- and time-dependent suppression of total ADPN secretion from 3T3-L1 adipocytes, while increasing total intracellular ADPN detected by immunoblots (Figure 1A-1D). Viability of the adipocytes was unaffected by these low concentrations of TSE (not shown). Inhibition of total ADPN secretion into the conditioned medium from TSE exposed 3T3-L1 adipocytes was confirmed by ADPN ELISA (Figure 1E). In addition, exposure of mouse primary adipocytes to 1.5% TSE ex vivo for 20 h significantly suppressed ADPN secretion, while increasing intracellular ADPN content (Figure 1F). Importantly, exposure of mice to TSE also induced a large decrease in plasma concentrations of total ADPN in vivo (Figure 1G), consistent with prior publications in smoke-exposed mice [11] and in human smokers [6,7]. Thus, smoke-induced retention of ADPN within adipocytes contributes to the decreased secretion of ADPN from adipose tissue and low plasma levels of ADPN in smokers [6,7,9,10].

### Tobacco smoke exposure traps HMW ADPN intracellularly

**Figure 2:**
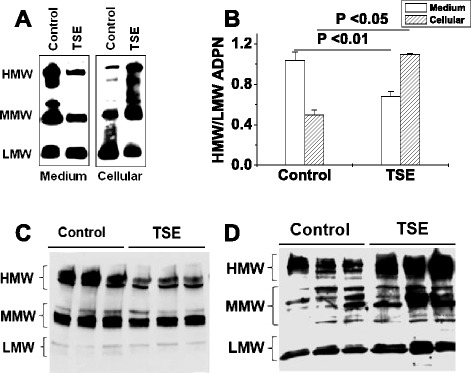
Tobacco smoke exposure traps HMW ADPN intracellularly. Panels **A,B**: Representative immunoblots of ADPN isoforms **(A)** and summary statistics for the ratio of HMW/LMW ADPN **(B)** in conditioned medium and cellular homogenates from 3T3-L1 adipocytes treated with 0% (control) or 1.5% TSE for 20 h. Isoforms of ADPN were separated by electrophoresis through 2–15% SDS-PAGE gradient gels under nonreducing and non-heat-denaturing conditions. Panel **B** displays means ± SEM, n = 4; P-values were computed using Student’s t-test*.* Panels **C**, **D**: Representative immunoblots showing the distribution of ADPN isoforms in plasma **(C)** and in epididymal adipose tissue **(D)** from wild-type mice after RPMI (control) or TSE injections.

Among the three different multimeric forms of ADPNs, HMW ADPN has been shown to be the most biologically active [4,5] in promoting insulin-induced glucose handling [3-6]. In the current study, we assessed the three major multimeric forms of ADPNs by immunoblots under non-reducing conditions [19]. We found that the decrease in total ADPN secretion from cultured 373-L1 adipocytes after TSE exposure (Figure 1A-E) was mainly attributable to decreased secretion of HMW ADPN (Figure 2A,B), accompanied by increased intracellular accumulation of HMW ADPN (Figure 2A,B). Likewise, we found that mice injected with TSE exhibited a loss of mainly HMW ADPN from plasma (Figure 2C) and HMW ADPN accumulated in their adipose tissue (Figure 2D).

### Tobacco smoke exposure dysregulates the expression of ADPN chaperones

**Figure 3:**
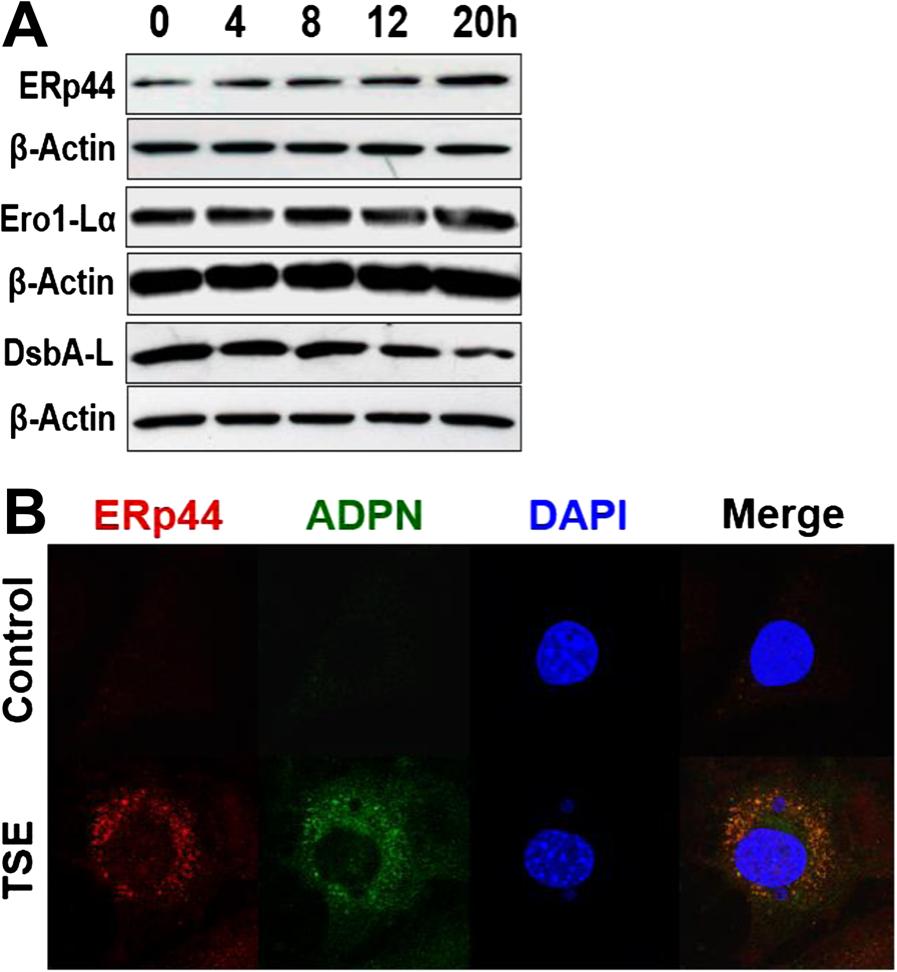
Tobacco smoke exposure dysregulates the expression of ADPN chaperones. Panel **A** shows representative immunoblots for time-dependent effects of TSE on the expression of ERp44, Ero1-Lα, and DsbA-L in 3T3-L1 adipocytes exposed to 1.5% TSE for 0-20 h. Panel **B** shows confocal fluorescent micrographs of representative 3T3-L1 cells that were stained simultaneously with anti-ERp44 (*red*) and anti-ADPN (*green*) antibodies, as well as DAPI (*blue*; nuclear stain). The yellow color in the merged images (*Merge*) demonstrates co-localization of ERp44 and ADPN in the ER around the nucleus.

Assembly and secretion of adiponectin oligomers from adipocytes is tightly regulated by thiol redox status in the ER through ERp44 and Ero1-Lα [20,21]. ERp44 is an ER resident chaperone that inhibits the secretion of ADPN through thiol-mediated retention, while Ero1-Lα releases HMW adiponectin from ERp44 [20,21]. In addition, DsbA-L has been shown to promote adiponectin multimerization and secretion [22,23]. In the current study, we found that TSE exposure of cultured adipocytes induced time- (Figure 3A) and dose- (not shown) dependent up-regulation of ERp44 and down-regulation of DsbA-L. TSE exposure, however, did not affect the amount of Ero1-Lα in adipocytes (Figure 3A), suggesting that the high intracellular levels of ERp44 would be unopposed. Additionally, our confocal microscopic analyses revealed that TSE exposure of adipocytes increased intracellular staining for ERp44 (red) and ADPN (green, Figure 3B). Importantly, this intracellular ADPN colocalized with ERp44 (yellow color in the merged images, Figure 3B), indicating ADPN accumulation in the ER, presumably physically associated with ERp44.

We conclude that tobacco smoke exposure suppresses ADPN secretion from adipocytes by specifically trapping HMW ADPN intracellularly, thereby contributing to decreased blood levels of ADPN in smokers. These results provide a novel mechanism for hypoadiponectinemia, which may help to explain impaired insulin-mediated glucose handling and the increased risk of T2DM in smokers.

## Abbreviations

ADPN: Adiponectin
DsbA-L: Disulfide-bond A oxidoreductase-like protein
ER: Endoplasmic reticulum
Ero1-Lα: ER oxidoreductase 1-Lα
ERp44: ER protein 44
TSE: Tobacco smoke extract
T2DM: Type 2 diabetes mellitus
HMW ADPN: High-molecular weight adiponectin
LMW ADPN: Low-molecular weight adiponectin
MMW ADPN: Medium-molecular weight adiponectin
